# Editorial: Digital technology in the management and prevention of diabetes

**DOI:** 10.3389/fendo.2024.1511236

**Published:** 2024-10-24

**Authors:** Yun Shen, Xiantong Zou, Gang Hu

**Affiliations:** ^1^ Chronic Disease Epidemiology Laboratory, Pennington Biomedical Research Center, Baton Rouge, LA, United States; ^2^ The Department of Endocrinology and Metabolism, Peking University People’s Hospital, Beijing, China

**Keywords:** digital technology, diabetes, management in health, telemedicine, remote rehabilitation

The rapid advancement of digital technologies has transformed the landscape of healthcare, particularly in the management and prevention of chronic diseases (1–3). Digital tools offer unprecedented opportunities to improve patient outcomes through better monitoring, individualized care, and more efficient communication between patients and healthcare providers. In this Research Topic, *Digital Technology in the Management and Prevention of Diabetes*, we bring together cutting-edge research exploring the application of digital tools in the field of diabetes care including 8 original research, 1 perspective article and 1 systematic review. These ten manuscripts featured in this Research Topic represent a diverse range of topics, from artificial intelligence-driven diagnostics to mobile health interventions, all of which highlight the promise of technology in revolutionizing diabetes care.

Specifically in this Research Topic, Lv et al. developed interpretable machine learning models based on three weighted diversity density (WDD)-based algorithms to diagnose type 2 diabetes mellitus (T2D) using physical examination indicators. The algorithms demonstrated strong predictive performance with inclusion of algorithms tolerant to missing values made the models robust and applicable for broader use in clinical settings. Peleg et al. developed and validated the questionnaire of digital tool use for diabetes to assess the type, number, and frequency of digital tools used by patients with T2D. A mixed-methods approach was used, including qualitative phone surveys of T2D patients, endocrinologists, and technology experts to develop the questionnaire, followed by a quantitative phase involving 367 participants. The questionnaire was found to be valid and reliable in identifying digital tools used for T2D self-management, despite variations in factor structures between ethnic groups. The study provided a standardized method for evaluating digital tool use and could be adapted for other illnesses by modifying specific instructions and wording. Matboli et al. developed a machine learning-based model to identify key features that influence drug responses in the treatment of T2D using medicinal plant-based drugs and a probiotics drug. The drugs effectively reduced liver inflammation, insulin resistance, and improved lipid profiles and kidney function biomarkers, particularly at higher doses. The machine learning model identified 13 molecular features, 10 biochemical features, and 20 combined features. This model highlighted the potential of machine learning in identifying therapeutic targets related to T2D pathogenesis. Yang et al. explored the impact of remote resistance exercise programs delivered via a smartphone application on skeletal muscle mass among elderly patients with T2D. After the intervention, there was a significant increase in skeletal muscle index (SMI) for both males (31.64 to 33.25 cm²/m²) and females (22.72 to 24.28 cm²/m²). Improvements were also observed in skeletal muscle cross-sectional area (SMA), radiodensity (SMD), and intermuscular adipose tissue (IMAT). The study demonstrated that remote resistance exercise programs delivered via smartphone are effective in increasing skeletal muscle mass in elderly patients with T2D. Yuan et al. also used the same platform to compare the effects of tele-rehabilitation with conventional face-to-face rehabilitation for patients with T2D and heart failure with preserved ejection fraction (HFpEF) in a real-world setting. A total of 90 patients using tele-rehabilitation were matched with 90 patients receiving face-to-face rehabilitation. Both groups showed significant improvements in short physical performance battery (SPPB) scores, 6-minute walk distance, gait speed, and quality of life (EQ-5D-5L) from 3 to 6 months after rehabilitation. There were no significant differences in functional outcomes or quality of life between the two groups. The study concluded that tele-rehabilitation is non-inferior to face-to-face rehabilitation and was well-accepted by patients, suggesting it could be a viable alternative to conventional rehabilitation programs among this specific population. Fundoiano-Hershcovitz et al. evaluated the relationship between depression, walking activity, and blood glucose (BG) levels in a cohort of 989 users with T2D and prediabetes by using the Dario digital health platform. Users with self-reported depression had higher BG levels compared to those without depression. Depression was significantly associated with a lower average number of steps per month, and the number of steps predicted the next month’s average BG adjusting for depression. Walking activity mediated the effect of depression on BG levels. These findings suggest that regular walking can help mitigate the negative impact of depression on BG control in individuals with T2D and prediabetes, supporting the integration of walking into treatment protocols as a simple and effective intervention. Su et al. compared the effects of different intensities of aerobic exercise delivered by a smartphone application-based program on glycemic control, pain relief, and functional outcomes in patients T2D and knee osteoarthritis (KOA) in a randomized trial setting. A total of 228 patients were randomized into three groups: high-intensity, moderate-intensity, and regular rehabilitation programs. After six months, the high-intensity group showed a significantly greater reduction in HbA1c levels compared to the other groups. However, while all groups saw improvements in pain and quality of life as measured by the KOOS subscales, there were no significant differences between the intensities of exercise in terms of pain relief or functional improvement. All groups also experienced reductions in BMI, but these reductions were not statistically different across the groups. The findings suggested that high-intensity aerobic exercise offers superior glycemic control but does not provide additional benefits over moderate-intensity or regular rehabilitation programs for pain and functional outcomes in KOA patients. Koçkaya et al. gathered opinions from specialized physicians on current diabetes management through an online questionnaire and in-person discussion sessions at the Diabetes Innovation Summit 2023. According to the respondents, around 60% of diabetes patients followed multiple daily injections (MDI), with 62% using blood glucose monitors (BGM), 31% using intermittent-scanning continuous glucose monitors (isCGM), and 23% using continuous glucose monitors (CGM). Physicians expressed concerns about misleading HbA1c results and challenges in achieving TIR targets despite CGM use. The present study highlights that physicians are generally supportive of utilizing new technology, while it is a long journey to improve the concepts of using technologies in public.

Shomali et al. wrote a piece of perspectives in this Research Topic. They explored critical elements of digital health innovations in diabetes and cardiometabolic care. They also highlighted that digital health technologies, such as CGM, wearable devices, and artificial intelligence (AI), provide real-time data that enable both patients and healthcare professionals to manage diabetes more effectively. The integration of these technologies into everyday life would allow for continuous, personalized care outside of traditional clinical settings. They demonstrated that these innovations enhance self-management, improve patient engagement, and offer more timely interventions, thus improving health outcomes for people living with diabetes.

The only one systematic review and meta-analysis in this Research Topic reviewed and analyzed the effects of whole-body vibration (WBV), a digital technology-based therapeutics on glycemic control in patients with T2D (Fabregat-Fernández et al.). Six randomized clinical trials involving 223 participants met the inclusion criteria for the systematic review, with four of those studies qualifying for the meta-analysis. The meta-analysis demonstrated a positive and significant effect size, indicating a substantial improvement in glycosylated hemoglobin levels among WBV-treated patients compared to control groups. The results suggested that WBV may be beneficial for improving glycemic control in T2D patients.

In conclusion, the manuscripts featured in this Research Topic highlight the transformative potential of digital technologies in diabetes care. However, challenges remain in achieving widespread adoption, particularly in overcoming financial and technological barriers. The findings underscore the need for continued research and the development of accessible, user-friendly technologies to meet the needs of diverse patient populations. As the field advances, digital health innovations are poised to play an increasingly critical role in the prevention and management of diabetes, paving the way for more efficient and equitable healthcare delivery.
